# Supercritical Fluid Synthesis and Tribological Applications of Silver Nanoparticle-decorated Graphene in Engine Oil Nanofluid

**DOI:** 10.1038/srep31246

**Published:** 2016-08-04

**Authors:** Yuan Meng, Fenghua Su, Yangzhi Chen

**Affiliations:** 1 School of Mechanical and Automotive Engineering, South China University of Technology, Guangzhou, 510640, P. R. China

## Abstract

Silver nanoparticle-decorated graphene nanocomposites were synthesized by a facile chemical reduction approach with the assistance of supercritical CO_2_ (ScCO_2_). The silver nanoparticles with diameters of 2–16 nm are uniformly distributed and firmly anchored on graphene nanosheets. The tribological properties of the as-synthesized nanocomposites as lubricant additives in engine oil were investigated by a four-ball tribometer. The engine oil with 0.06~0.10 wt.% Sc-Ag/GN nanocomposites displays remarkable lubricating performance, superior than the pure engine oil, the engine oil containing zinc dialkyl dithiophosphate (ZDDP), as well as the oil dispersed with the single nanomaterial of graphene oxides (GOs) and nano-Ag particles alone. The remarkable lubricating behaviors of Sc-Ag/GN probably derive from the synergistic interactions of nano-Ag and graphene in the nanocomposite and the action of the formed protective film on the contact balls. The anchored nano-Ag particles on graphene expand the interlamination spaces of graphene nanosheets and can prevent them from restacking during the rubbing process, resulting in the full play of lubricating activity of graphene. The formed protective film on the friction pairs significantly reduces the surface roughness of the sliding balls and hence preventing them from direct interaction during the sliding process.

Recently, tremendous research efforts have been devoted to employing nanomaterials as lubricating oil additives thanks to their facile preparation, large specific surface area and tiny nano-size dimension^1^,^2^,^3^,^4^. A variety of nanostructured metals and metallic oxides, including copper, nickel, silver and their oxides^5^,^6^,^7^,^8^,^9^,^10^,^11^ have been reported to be excellent additives in liquid lubricants. Chen *et al*.^7^ demonstrated that nickel-based poly-alpha-olefin (PAO6) nano-lubricant exhibited good antiwear behaviors owing to the adsorption and deposition effects of the active nickel cores. Gusain *et al*.^9^ found that CuO nanorods in ionic liquids possessed good friction-reduction (15~43%) and antiwear abilities (26~43%) in comparison with the PEG 200 and 10w40 engine oil. It has been documented that nano-sized metals and their corresponding oxides can not only form compact protective layers easily^5^,^6^,^7^,^8^,^9^, but also have rolling effects^10^,^11^, both of which can lead to effective friction and wear reductions.

Graphene and its derivatives have been considered as one of the most promising and attractive lubricating nanomaterials and hold potentials in tribological applications. They were employed not only as solid lubricants to form protective membranes on certain substrates^12^,^13^,^14^, but also as reinforced phases in various polymers^15^,^16^, ceramics^17^,^18^ and lubricating additives^19^,^20^,^21^ in various base oils. Won *et al*.^12^ demonstrated that significant friction reduction was achieved via depositing graphene coatings on Cu substrate by chemical vapor deposition (CVD). Li *et al*.^15^ proved that the loading of GO in nitrile rubber (NBR) matrix dramatically reduced the friction coefficient and specific wear rate under dry sliding and water-lubricated conditions. Significant improvements in the friction reduction and wear resistance were achieved through the introduction of 0.025 mg·mL^−1^ ultrathin graphene in engine oil, as reported by Eswaraiah *et al*.^19^ These excellent tribological performances of graphene are attributed to its extremely thin laminated structure and remarkable mechanical strength, which can lower shear stress and prevent direct interaction between contact pairs.

Notably, because of the unique two dimension structure and high specific surface area, graphene and its derivatives have been extensively employed as ideal substrates for fabricating functional composites^22^,^23^,^24^. A great deal of graphene-based composites incorporated with nano-sized metals or metallic oxides have been prepared for certain applications^25^,^26^,^27^,^28^. However, to date, few studies have been involved in their tribological applications^29^,^30^ and it is still rather challenging to achieve optimal combination for those base ingredients at nanoscale dimension. Supercritical fluid technique has been demonstrated to be effective and efficient technique for synthesizing high quality nanocomposites^31^,^32^,^33^,^34^,^35^,^36^. Supercritical carbon dioxides (ScCO_2_) possess readily accessible supercritical conditions and various unique properties including gas-like diffusivity, extremely low viscosity, excellent mass-transfer activity and near-zero surface tension^35^,^36^, which are beneficial for wetting the substrates and loading the metal nanoparticles onto the substrate surfaces uniformly. Therefore, nanocomposites fabricated by the ScCO_2_ technique usually have more well-defined microstructures and exhibit better macro-properties^37^,^38^,^39^. For example, Zhao *et al*.^37^ prepared PtRu alloy nanoparticle-decorated graphene composites using supercritical fluid method. Utrafine PtRu nanoparticles were uniformly distributed on graphene surface and the composites exhibited much higher electrocatalytic activity and stability than PtRu/carbon black.

In this work, silver nanoparticle-decorated graphene nanocomposites were successfully synthesized in ScCO_2_ fluids by a facile chemical reduction approach. The resulting composites (Sc-Ag/GN) were fully characterized by X-ray diffraction (XRD), thermogravimetric analysis (TGA), X-ray photoelectron spectroscopy (XPS), transmission electron microscopy (TEM), as well as other techniques. The lubricating performances of the nanomaterials as lubricant additives in 10w40 engine oil were investigated by a standardized four-ball test method. For the sample of Sc-Ag/GN, ultrafine silver particles (2–16 nm) were evenly dispersed on graphene sheets. Sc-Ag/GN as additives in 10w40 engine oil displays the optimal friction-reducing and antiwear abilities compared to the single additives of GO, silver nanoparticles and the commercial ZDDP. By analyzing the wear scar surfaces, the possible friction-reducing and antiwear mechanisms of Sc-Ag/GN composites in engine oil have been proposed. The findings here provide an efficient approach for fabricating graphene/nano-metal composites with remarkable tribological properties readily for potential industrial applications.

## Results

### Structure and Morphology

**Figure 1:**
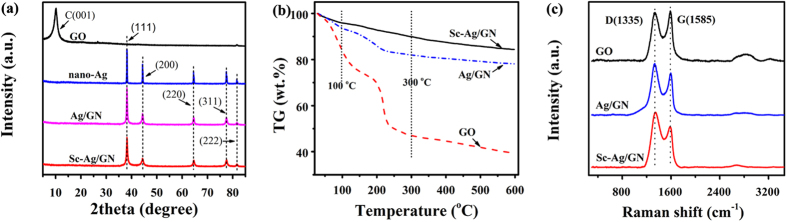
(**a**) XRD patterns of GO, nano-Ag, Ag/GN and Sc-Ag/GN; (**b**) TGA curves and (**c**) Raman spectra of GO, Ag/GN and Sc-Ag/GN.

Figure 1a shows the XRD patterns of GO, nano-Ag, Ag/GN and Sc-Ag/GN. The Ag/GN nanocomposite is prepared by the same protocol as Sc-Ag/GN but without the introduction of ScCO_2_ during deposition process. The typical diffraction peak at 10.4° corresponds to the (001) crystal plane of GO. Such strong signal disappears completely in the patterns of Ag/GN and Sc-Ag/GN, which is probably attributed to the fully exfoliation of the graphene nanosheets^32^. We postulate that the anchored nano-Ag particles in the nanocomposites exfoliate the graphene nanosheets and prevent them from restacking orderly. In the case of nano-Ag pattern, the peaks at 38.1°, 44.2°, 64.4°, 77.4°, 81.5° are ascribed to its (111), (200), (220), (311) and (222) crystallographic planes (*JCPDS No.04-0783*), respectively. These peaks are broadened in the Ag/GN and Sc-Ag/GN samples, which is ascribed to the decreased grain sizes of the anchored Ag nanoparticles. Encapsulated by graphene nanosheets, the grain growth of the silver cores is perhaps restricted during the crystallization process.

Figure 1b exhibits the TGA curves of GO, Ag/GN and Sc-Ag/GN. The weight loss below 100 °C is attributed to the evaporation of adsorbed water. The most weight loss of GO below 100 °C is because a large number of water molecules are encapsulated by a multitude of oxygen-containing groups on its surface. The main weight loss of the three samples in the range of 100~300 °C originates from the thermal decomposition of the oxygen-containing groups^31^. When compared to GO, the nanocomposites of Ag/GN and Sc-Ag/GN exhibit less weight loss from 100 to 300 °C, because most of oxygen-containing groups have been reduced by glucose during the deposition process. Meanwhile, the different weight loss between Sc-Ag/GN and Ag/GN from room temperature to 600 °C suggests that the two samples have different amounts of nano-Ag particles. The unique properties such as gas-like diffusivity, extremely low viscosity and excellent mass-transfer activity of ScCO_2_ are beneficial for transferring more precursors of silver nitrate onto graphene surfaces^37^,^38^, resulting in more nano-Ag particles anchored on graphene nanosheet surfaces.

Raman spectroscopy is employed to further elucidate the microstructures of GO, Ag/GN and Sc-Ag/GN. As shown in Fig. 1c, two characteristic peaks at 1335 cm^−1^ and 1585 cm^−1^ are ascribed to the D band (*κ*-point phonon of *A*_*1g*_ symmetry) and G band (*E*_*2g*_ phonon of C sp^2^ atoms) of GO^33^, respectively. The intensity ratios of D band *vs.* G band (I_D_/I_G_) in the nanocomposites are much higher than that in GO, which is the result of the massive decorations of nano-Ag particles on graphene surfaces^34^. In addition, it has been recorded that the electromagnetic and chemical enhancement effect, that is caused by decorated Ag nanoparticles in this work, can strengthen the D and G band intensity of the nanocomposites^26^,^40^. No other peaks below 1000 cm^−1^ are observed, which indicates that no impurity of metallic oxides in Ag/GN and Sc-Ag/GN is present.

**Figure 2:**
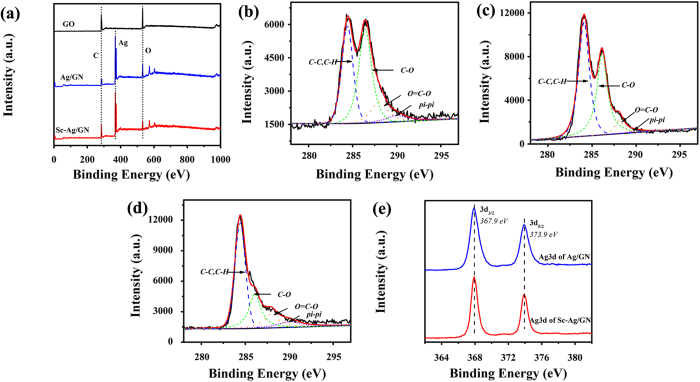
(**a**) XPS survey scans of GO, Ag/GN and Sc-Ag/GN; C_1s_ XPS spectra of (**b**) GO, (**c**) Ag/GN, (**d**) Sc-Ag/GN; (**e**) High resolution Ag_3d_ spectra of Ag/GN and Sc-Ag/GN.

XPS spectra of the GO, Ag/GN and Sc-Ag/GN are shown in Fig. 2. The weakened peaks of oxygen and the decreased C/O atomic ratios of Ag/GN and Sc-Ag/GN are observed in Fig. 2a, suggesting that the GO is reduced to graphene in the nanocomposites^41^. Meanwhile, the much higher carbon-oxygen atomic ratio (C/O) in Sc-Ag/GN (4.5/1) than that in Ag/GN (2.3/1) demonstrates that ScCO_2_ favors the reduction of GO during the synthetic process. The C_1s_ spectrum of GO (Fig. 2b) can be fitted into four curves centered at 284.07 eV (C-C,C-H), 286.15 eV (C-O), 288.08 eV (O=C-O) and 290.00 eV (*pi-pi*), respectively. The differences of C1s spectra between Fig. 2c,d indicate that the introduction of ScCO_2_ changes the chemical composition of the produced nanocomposite. Compared to that in Sc-Ag/GN (Fig. 2d), higher peak intensity of C-O in the Ag/GN (Fig. 2c) observed further confirms the reinforced reduction effect of ScCO_2_. The high resolution electron spectra of Ag_3d_ in Ag/GN and Sc-Ag/GN (Fig. 2e) prove that silver element in nano-Ag particles exist in zero valence state. No other signals from Ag with different charge states are detected.

**Figure 3:**
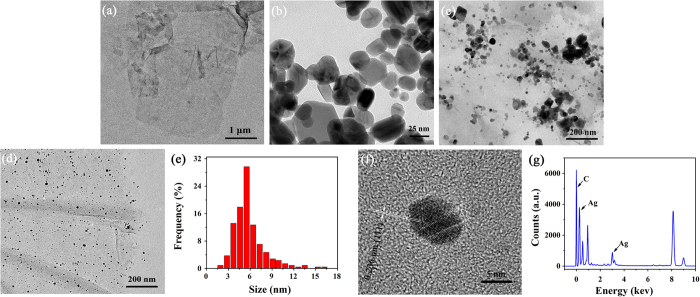
TEM micrographs of (**a**) GO, (**b**) nano-Ag, (**c**) Ag/GN and (**d**) Sc-Ag/GN; (**e**) Size distribution of silver nanoparticles in Sc-Ag/GN; (**f**) HR-TEM micrograph of single silver particle in Sc-Ag/GN; (**g**) EDS of Sc-Ag/GN.

The morphologies of GO sheet, nano-Ag particles, Ag/GN and Sc-Ag/GN are then examined by TEM measurements and shown in Fig. 3. GO has thin and transparent sheets with tiny wrinkles on its surface (Fig. 3a). Figure 3b displays that nano-Ag particles possess near-spherical structures and wide size distributions with diameters ranging from 10 nm to 100 nm. As the grain size decreases, the shape becomes better defined. The influences of ScCO_2_ on the morphologies and microstructures of the as-prepared nanocomposites are revealed by comparing the TEM images of Ag/GN and Sc-Ag/GN. As shown in Fig. 3c, polydisperse Ag particles with irregular shapes and morphologies are presented in the sample of Ag/GN, along with wide size distributions and appearance of even large massive clusters. In contrast, the spherical Ag particles with tiny nano-size dimension are evenly anchored on graphene nanosheet surfaces in the Sc-Ag/GN (Fig. 3d). Owing to a variety of excellent characteristics including enhanced transport coefficient, gas-like viscosity and almost zero surface tension, ScCO_2_ favors the exfoliation of graphene nanosheets, enhances the adhesion and dispersion of silver precursors on the nanosheet surfaces, and prevents the wild growth of the silver cores during the synthetic process^30^,^38^. As a result, the Ag nanoparticles with smaller grain size and high uniformity are achieved for the sample of Sc-Ag/GN. The size distribution of Ag nanoparticles in the Sc-Ag/GN is shown in Fig. 3e. It follows a parabolic pattern ranging from 2 nm to 16 nm and dominates in the range of 3–9 nm. HR-TEM image of one nano-Ag particle taken from Fig. 3d is presented in Fig. 3f. The lattice spacing of 0.236 nm corresponds to (111) lattice plane of typical FCC Ag crystal. Figure 3g shows EDS spectrum of Sc-Ag/GN from Fig. 3d. The strong silver and carbon peaks derive from silver particles and graphene in the Sc-Ag/GN, respectively. Other peaks are from the copper grid used to load the testing samples.

**Figure 4:**
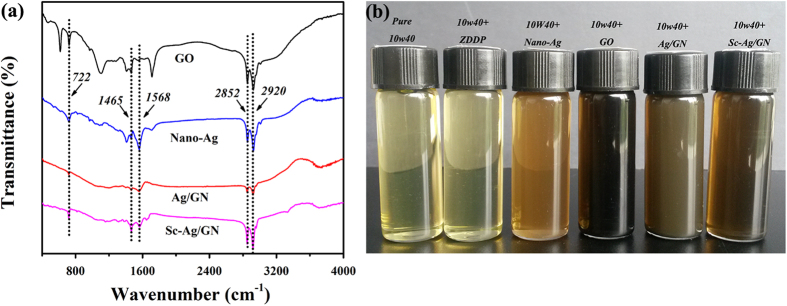
(**a**) FT-IR spectra of the modified GO, nano-Ag, Ag/GN and Sc-Ag/GN by stearic amine; (**b**) Photographs of the modified nano-oils after resting for two weeks.

It is well known that the dispersibility and stability of nanomaterial additives in oil greatly affect the lubricating performances of the dispersed oil. Fourier transform infrared (FT-IR) spectra of the modified additives (GO, nano-Ag, Ag/GN and Sc-Ag/GN) by stearic amine are shown in Fig. 4a. Two characteristic peaks at 2920 cm^−1^ and 2852 cm^−1^ from the C-H bond stretching in -CH_2_- and -CH_3_- groups are observed, and the peak at 722 cm^−1^ is ascribed to the C-H bond vibration in -(CH_2_)_n_- long chains from stearic amine. The results indicate that long alkyl chains of stearic amine have been grafted onto the nanomaterial additives. The grafting dramatically increases the dispersibility and stability of the modified additives in engine oil, evidenced by the static tests of dispersed nano-oils. Figure 4b shows that the dispersed nano-oils with these modified nano-additives maintain stable without precipitates formed after resting for two weeks.

### Lubrication Performances

**Figure 5:**
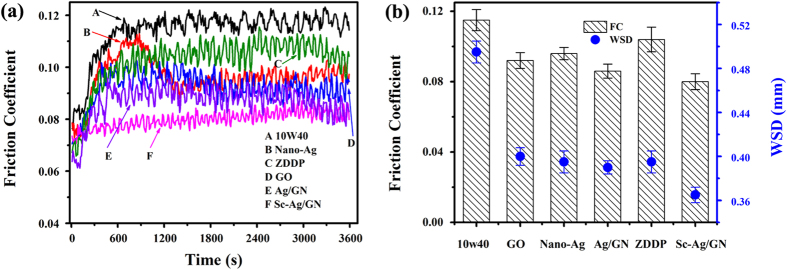
(**a**) Friction coefficient as a function of the sliding time for pure 10w40 and different nano-oil samples; (**b**) Average friction coefficient and wear scar diameter (WSD) for pure 10w40 and different nano-oil samples.

The commercial antiwear and extreme-pressure additive of ZDDP was selected as a reference to compare its lubrication performance with the as-prepared nanomaterial additives. Figure 5a shows the typical friction coefficient curves of the different oil samples (bare 10w40, 10w40+0.10 wt.%ZDDP, 10w40+0.10 wt.% nano-Ag, 10w40+0.10 wt.% GO, 10w40+0.10 wt.% Ag/GN, 10w40+0.10 wt.% Sc-Ag/GN) as a function of the sliding time. The friction coefficient of the bare engine oil reaches 0.12 at around 15 mins of sliding and then maintains at this level till the end of the test. The oil samples dispersed with Ag/GN, ZDDP and GO display the similar trend with the bare oil but the highest friction coefficients are decreased. In the case of the nano-Ag dispersed oil, its friction coefficient reaches 0.11 at around 12 mins of sliding, gradually decreases to 0.09 at around 20 mins of sliding, and then maintains at this level till the end. The friction coefficient of the Sc-Ag/GN dispersed nano-oil slightly increases at the beginning, but the amplitude is quite small (from 0.0703 to 0.0804) as compared with other additives. As expected, this sample shows the lowest and most steady friction coefficient with very little fluctuations during the sliding process.

The average friction coefficients and wear scar diameters (WSDs) of these oil samples are shown in Fig. 5b. Obviously, the friction coefficients and WSDs decrease upon the introduction of all the additives. The Sc-Ag/GN dispersed engine oil exhibits the lowest friction coefficient and WSD, followed by the Ag/GN dispersed one. The superior friction-reducing and antiwear abilities of the nanocomposites than the single GO, nano-Ag and even ZDDP may result from the synergistic lubricating behaviors^42^ between Ag nanoparticles and graphene nanosheets in the composites. Moreover, it can be seen that the lubricating performance of Sc-Ag/GN is better than that of Ag/GN, which is ascribed to their different microstructures and morphologies. The smaller grain size and more uniform dispersion of nano-Ag particles anchored on graphene surfaces may favor the release of the friction-reducing and antiwear potentials of nano-Ag and graphene nanosheets in the as-prepared nanocomposite.

**Figure 6:**
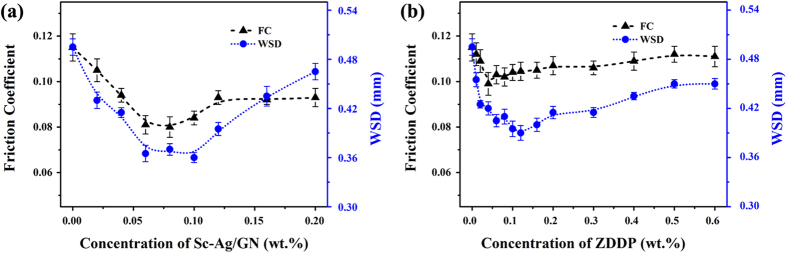
Friction coefficient and WSD versus concentration of (**a**) Sc-Ag/GN and (**b**) ZDDP dispersed in 10w40 oil (343 N, 1200 rpm, 60 mins, 75 ± 1 °C).

The concentration influences of the commercial ZDDP and the Sc-Ag/GN additives on the friction coefficients and WSDs are shown in Fig. 6. As shown in Fig. 6a, the curves of friction coefficient and WSD *vs.* Sc-Ag/GN concentration display “V” shape and have a narrow flat bottom where the lowest friction coefficient and WSD are presented. At the bottom point, the friction coefficient and WSD are reduced by 30.4% and 27.4% respectively, compared with bare engine oil. Beyond this bottom, the friction coefficient and WSD gradually increase with the increasing of Sc-Ag/GN concentration. The optimal Sc-Ag/GN concentration in engine oil is 0.06~0.10 wt%. Excessive amount of Sc-Ag/GN in oil may affect the dispersion level of the nanocomposites and the homogeneousness of the dispersed nano-oil. In addition, Zhang *et al*.^43^ confirmed that excessive graphene added in oil caused the stacking of graphene between friction pairs that blocked the oil film hence detrimental to the lubricating performances. Figure 6b displays the friction coefficient and WSD variations with increasing ZDDP concentration in engine oil. The lowest friction coefficient (FC) is obtained as 0.04 wt.% ZDDP is added in the engine oil. With the further increasing of ZDDP, the friction coefficient increases slightly less than 10%. In contrast, the WSD decreases dramatically with increasing ZDDP concentration up to 0.10 wt.% and then increases rapidly with the further increasing of ZDDP concentration in oil. Note that, the ZDDP dispersed oil show the most significant reduction of 11.6% for friction coefficient and 20.2% for WSD. When the same concentrations are employed, the nanomaterial of Sc-Ag/GN outperforms the commercial ZDDP to endow the dispersed oil with superior friction-reducing and antiwear abilities.

### SEM and XPS Analyses of Wear Scar Surfaces

**Figure 7:**
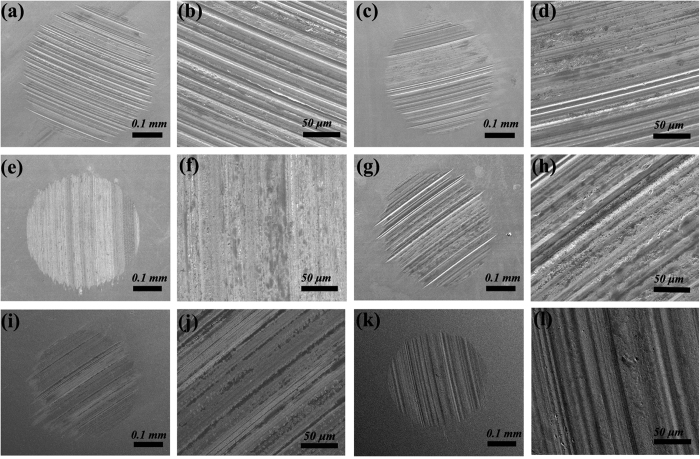
SEM images of the wear surfaces lubricated by various oils (343 N, 1200 rpm, 60 mins, 75 ± 1 °C). (**a,b**) Pure 10w40, (**c,d**) 10w40+0.10 wt.% GO, (**e,f**) 10w40+0.10 wt.% nano-Ag, (**g,h**) 10w40+0.10 wt.% ZDDP, (**i,j**) 10w40+0.10 wt.% Ag/GN, (**k,l**) 10w40+0.10 wt.% Sc-Ag/GN.

Figure 7 shows the representative SEM images of the wear surface of the steel balls lubricated by the bare 10w40 engine oil and the different dispersed oils. It is worth noting that a relatively large WSD with severe scuffing and adhesion wear is observed on the ball lubricated by the bare oil, as shown in Fig. 7a,b. The addition of graphene in oil reduces the WSD (Fig. 7c), but deep scratches and grooves are also observed on the wear surface (Fig. 7d). The WSD lubricated by the nano-Ag dispersed oil (Fig. 7e) is almost the same with the one lubricated by the graphene dispersed oil. However, the wear surface becomes much brighter and smoother and it seems to be covered by the deposited silver film, as shown in Fig. 7e,f. The furrows on this wear surface also become flat and smooth (Fig. 7f). The results suggest that the nano-Ag particles penetrate the friction interface and deposit on the contact pairs, which agrees well with the previous report^44^. As shown in Fig. 7g,h, the wear surface of the oil dispersed with ZDDP is also rough and shows deep grooves and severe scuffing although the WSD is reduced to around 0.395 mm. As expected, the ball lubricated by the oil dispersed with the nanocomposite (Ag/GN or Sc-Ag/GN) exhibits smaller WSD than the one lubricated by other oils, as shown in Fig. 7i,k. And their wear surfaces possess less and shallower furrows and scratches (Fig. 7i,l). Especially for the ball lubricated by the Sc-Ag/GN dispersed oil, its WSD around 0.360 mm is the smallest among all the samples and the wear surface is very smooth (Fig. 7k,l). Previous studies have documented that graphene and nanoparticle as lubricant additives can deposit on the friction interface and form a protective film^7^,^13^,^29^. In this work, we believe that graphene and nano-Ag from the nanocomposite deposit on contact ball surfaces during sliding process, and then form a protective film induced by the friction heat that can smooth the surfaces and reduce the rubbing between the balls. The hypothesis can be verified by the following results discussed next.

**Figure 8:**
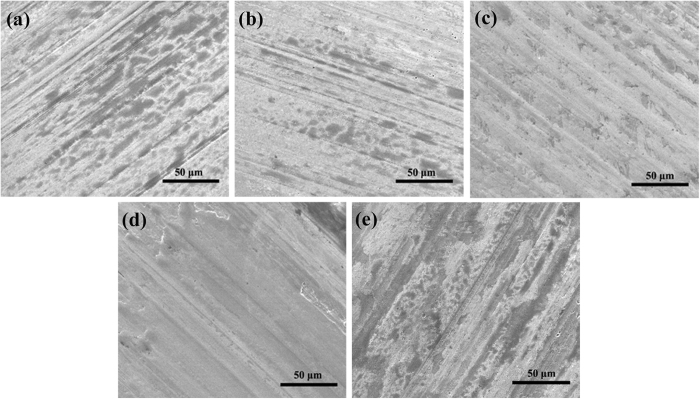
SEM images of the wear surfaces lubricated by 10w40+0.1 wt.% Sc-Ag/GN nano-oil after sliding for different times (343 N, 1200 rpm, 75 ± 1 ^o^C). (**a**) 5 mins, (**b**) 10 mins, (**c**) 20 mins, (**d**) 40 mins, (**e**) 60 mins.

The morphology evolution of the wear surface lubricated by the 0.10 wt.% Sc-Ag/GN dispersed nano-oil is illustrated in Fig. 8. The morphologies demonstrate great changes when increasing the rubbing time. On one hand, the scratches and grooves on the wear surface become more and more clear with increasing rubbing distance, as shown in the SEM images from Fig. 8a–e. It might be attributed to the strengthening abrasive wear with the increasing of sliding time. On the other hand, the as-formed deposition film on the ball varies with the increasing of sliding time. After sliding for 5 and 10 mins, a thick and discontinuous deposition film is observed on the wear surfaces (Fig. 8a,b). With increasing sliding time, the formed deposition film becomes thin, continuous and easily distinguishable, as shown in Fig 8c–e. As shown in Fig. 8e, it seems that most areas of the wear surface are covered with a thin deposition film after sliding for 60 mins. The transformation (e. g. from thick to thin, from discontinuous to continuous) of the deposition film has relation with the tribo-chemical reactions occurred onto the friction interfaces with the increase of sliding time. Such compositional evolution of the wear surface versus time was further subjected to XPS analysis.

## Discussion

**Figure 9:**
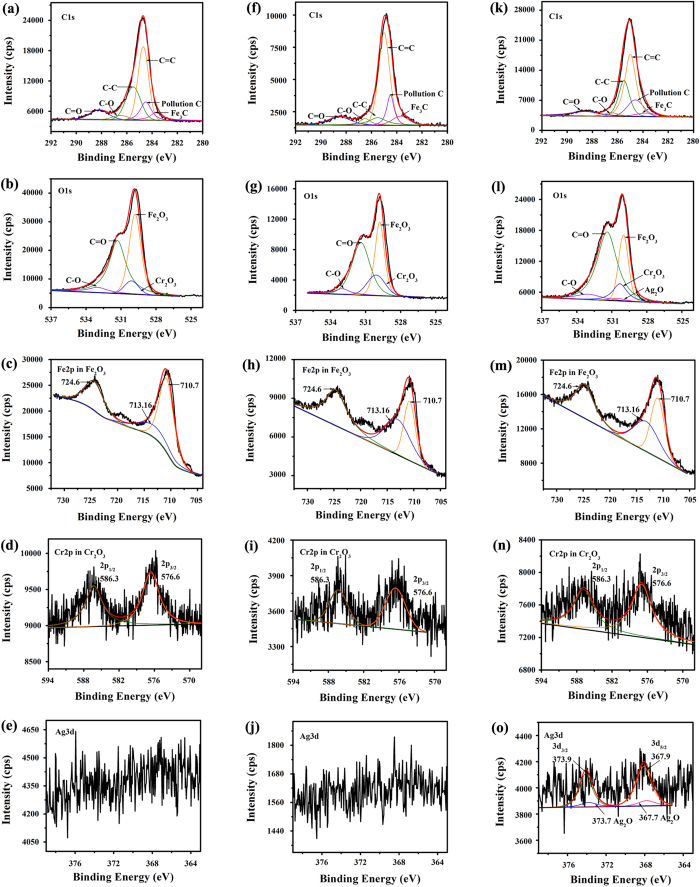
Curve-fitted XPS spectra of typical elements (C_1s_, O_1s_, Fe_2p_, Cr_2p_, Ag_3d_) on the wear surface lubricated by 10w40+0.1 wt.% Sc-Ag/GN nano-oil after sliding for different times (343 N, 1200 rpm, 75 ± 1 °C). (**a–e**) 5 mins, (**f–j**) 40 mins, (**k–o**) 60 mins.

Figure 9 shows the XPS spectra of C_1s_, O_1s_, Fe_2p_, Cr_2p_ and Ag_3d_ electron of the wear surface lubricated by the 0.10 wt.% Sc-Ag/GN dispersed oil after sliding at 343 N for a series of sliding times (5, 40, 60 mins). The C_1s_ spectra with six sub-peaks at 283.7, 284.6, 284.5, 285.5, 286.9 and 288.2 eV corresponds to graphene and its oxides, cementite and some polluted carbon on the wear surfaces^12^,^13^,^14^. Close examination on the three C_1s_ spectra in Fig. 9a,f,k reveals that remarkable signal changes are observed from the C-C and C=C sub-peaks. The intensity of C-C peak decreases firstly, and then increases with increasing sliding time, while the C=C peak exhibits the opposite trend. The result suggests that the graphene in Sc-Ag/GN probably undergoes two successive processes: (i) deposition on interface and (ii) rupture by stress and heat. The O_1s_ peak in all the samples can be de-convoluted into four sub-peaks around 530, 530.3, 531, 533 eV, indicating that Fe, Cr and C are partially oxidized during sliding. The constant increasing of C=O peak intensity is probably attributed to the continuous deposition and subsequent oxidation of graphene. Notably, the O_1s_ sub-peak from Ag_2_O is very weak, only can be observed after sliding for 60 mins (Fig. 9l). The result demonstrates that the encapsulated Ag nanoparticles by graphene are exposed on the friction interface and partially oxidized after sliding till 60 mins. The Fe_2p_ and Cr_2p_ spectra show the same chemical charge states after sliding for different times. The peak positions of Fe_2p_ at 710.7, 713.2 and 724.6 eV correspond to Fe_2_O_3_ (O_1s_ 710.7, 724.6 eV)^6^,^7^ and Fe_3_O_4_ (O_1s_ 713.2 eV)^6^,^7^. The Cr_2p_ spectra at 576.6 eV and 586.3 eV are attributed to Cr_2p3/2_ and Cr_2p1/2_ of Cr_2_O_3_^6^,^7^, respectively. The XPS spectra of Ag_3d_ on the wear surfaces after different sliding times are shown in Fig. 9e,j,o. The Ag_3d_ spectra with only noise signals in Fig. 9e,j imply that almost no Ag can be detected on the wear surfaces after 5 and 40 mins sliding, which might be due to the discontinuous deposited film formed onto the sliding balls (Fig. 8a–c) after sliding for insufficient time and the deposited Ag nanoparticles are encapsulated by graphene. The two weak peak signals at 373.9 (Ag_3d3/2_) and 367.9 eV (Ag_3d5/2_) are observed in the Ag_3d_ spectrum (Fig. 9o), suggesting that a small amount of Ag nanoparticles are exposed on the wear surface after 60 mins sliding. The other two weak sub-peaks at 367.7 and 373.7 eV with slight positive energy shift suggest that a few silver atoms in the particles are probably oxidized after sliding for 60 mins.

Table 1 gives the relative atomic contents of C, O, Fe, Cr, and Ag on the wear surface after different sliding times at 343 N. After sliding for 5 mins, a large amount of carbon is detected on the wear surface while no Ag is observed. Clearly, most of C is from the graphene in deposited Sc-Ag/GN that has formed a thick and discontinuous film on the wear surface (Fig. 8a). Instead, as the Sc-Ag/GN in the thick and discontinuous film has well-integrated structures and the Ag nanoparticles are fully encapsulated by graphene, it is reasonable that Ag atoms are not detected on the wear surface due to the ultrathin detecting depth (0.5–2.5 nm) of XPS analysis. Interestingly, the similar detection result with 5 mins is shown for the wear surface after sliding for 40 mins, which suggests that the well-integrated structures of Sc-Ag/GN are not destroyed by the friction force till the sliding for 40 mins. As the sliding time increases to 60 mins, the relative atomic content of C increases, while those of O, Fe and Cr decrease, and trace amount of Ag is detected. We postulate that a tribochemical reaction probably occurs onto the friction interface. The produced friction heat after sliding over 40 mins gradually rupture the well-defined structure of graphene and trigger the tribochemical reaction. Consequently, Ag nanoparticles are exposed to the friction interface and hence are detected. Meanwhile, such tribochemical reaction leads to the formation of a thin and uniform protective film (Fig. 8e), as well as the decreases of Fe and Cr contents on the wear surface.

**Figure 10:**
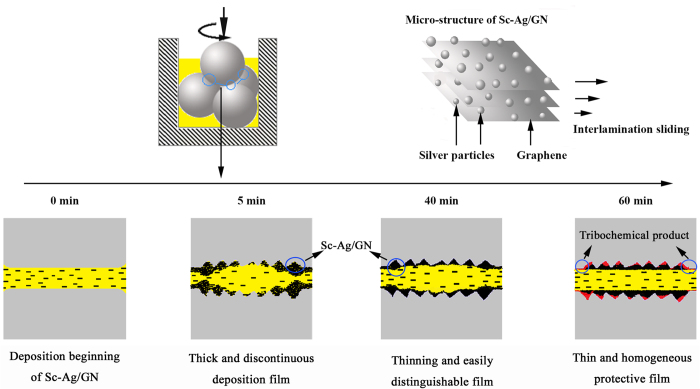
Schematic of the tribological model of 0.1 wt.% Sc-Ag/GN dispersed nano-oil.

The tribological process and proposed mechanism are illustrated by Fig. 10. As the sliding is started, the steel surfaces scratch each other because of the friction force, resulting in many pits and grooves (Fig. 7a) on the wear surface and the abrasive particles produced in oil. After the first sliding for 5 mins, the nanocomposites begin to deposit and accumulate in these pits and grooves till these defects are completely filled up. The formed deposition film is very thick and discontinuous (Fig. 8a). With the increasing of the sliding time, the deposition film gradually becomes thin and continuous because of the high pressure and squeezing action caused by the contact balls (Fig. 8a–e). As the sliding is time increased to 40 mins, the whole interface becomes smooth and the surface roughness is reduced distinctly (Fig. 8d). But the well-integrated structures of Sc-Ag/GN are not destroyed by the friction force, evidenced by XPS results (Fig. 9j and Table 1). With the further increasing of sliding time over 40 mins, the produced friction heat gradually rupture the well-defined structure of graphene and trigger the tribochemical reaction on the friction interface, resulting in the thin and uniform protective film completely formed (Fig. 8e). The formed protective film can prevent the friction pairs from direct contact and reduce the roughness of the surface^19^,^20^,^21^. Moreover, the deposited Sc-Ag/GN can absorb the oil due to its lipophilicity, which may favor stabilizing the lubricating film.

Analyzing the changes of friction coefficient (FC) in the sliding process is important to reveal the mechanism, and FC can be calculated by the following equation^43^:


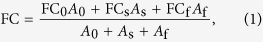


where *A*_0_ is the friction area of oil between friction pairs and Sc-Ag/GN, *A*_s_ is the dry-contact area of Sc-Ag/GN and *A*_f_ is the dry-contact area of furrows. The deposition film induced by Sc-Ag/GN protects the metal surfaces of contact pairs, resulting in the decrease of *A*_f_ and the increase of *A*_0_. The anchored nano-Ag particles on graphene sheets prohibit the restacking of graphene^38^,^40^, leading to the reduction of *A*_s_. Therefore, FC is markedly reduced at lower concentrations of Sc-Ag/GN compared with that in bare engine oil.

In addition, the base ingredients of Sc-Ag/GN (silver particles and graphene nanosheets) both have good lubricating capabilities and they can work cooperatively^29^,^30^,^42^. The decorated silver particles can exfoliate the graphene nanosheets which significantly facilitate the interlamination sliding. Due to the high mechanical strength, the exfoliated graphene nanosheets can undertake and distribute the loading effectively. Meanwhile, the “soft” Ag nanoparticles from the nanocomposites deposited on the wear interface can also exert their self-repairing properties^6^,^8^ when the sliding time is increased to over 40 mins.

In summary, the nanocomposite of graphene anchored with silver nanoparticles (Sc-Ag/GN) has been successfully prepared by a facile chemical reduction with the assistance of supercritical carbon dioxides (ScCO_2_). The anchored silver particles possess a narrow grain size range with diameters of 2–16 nm and uniform distribution on graphene sheets without aggregations, thanks to the unique properties of ScCO_2_. After simply modified by stearic amine, Sc-Ag/GN is successfully employed as lubricant additives in 10w40 engine oil. The friction coefficient and wear scar diameter are reduced up to 30.4% and 27.4% respectively, when 0.06~0.10 wt.% Sc-Ag/GN are dispersed in bare engine oil. The friction-reducing and antiwear abilities of Sc-Ag/GN are superior to single nano-silver, single graphene oxide, ZDDP, as well as the Ag/GN nanocomposite produced in air. During the sliding process, the Sc-Ag/GN deposits on the wear surface and forms a protective film that smoothes the contact surface and reduces the surface roughness of the contact pairs. The Sc-Ag/GN absorbs and stabilizes the oil film due to its lipophilicity and desirable surface microstructure. Moreover, the silver particles and graphene nanosheets of Sc-Ag/GN can work cooperatively to form the synergistic effect, which is another crucial factor for the super lubricating performances of Sc-Ag/GN in nano-oil. The findings here are very important and extremely valuable, not only a new type of excellent lubricating materials are obtained, but also may shed light on preparing nanoparticle based nanocomposites with remarkable tribological properties readily for potential industrial applications.

## Methods

### Chemicals

Glucose (C_6_H_12_O_6_) and silver nitrate (AgNO_3_) were supplied from Shanghai Richjoint Chemical Reagent Co., Ltd. Aqueous ammonia (NH_3_∙H_2_O) was purchased from Guangzhou Chemical Reagent Factory. Natural graphite (average size ~44 μm) was supplied from Qingdao Dongkai Co., Ltd. Sodium dodecyl sulfate (SDS) was supplied from Tianjin Fuchen Chemical Reagent Factory. Stearic amine (C_18_H_39_N) was supplied from Tianjin Guangfu Fine Chemical Research Institute. Ethanol was purchased from Tianjin Fuyu Fine Chemical Co., Ltd. All the chemicals were analytical grade and deionized water by distillation was used.

### Preparation of silver-decorated graphene composites

Graphene oxides (GO) was prepared from natural graphite powders by a modified Hummers method^45^,^46^ and employed as substrate to load silver particles. Silver-ammonia solution was obtained by dripping aqueous ammonia (1 mL, 25 wt.%) into silver nitrate solution (2 mL, 9.1 wt.%). GO ethanol suspension was prepared by ultrasonically dispersing graphene oxides in ethanol (50 mL) for 15 mins using a KQ-100D ultrasonicator (Dongguan, China). Typically, SDS solution (1 mL, 5 mg·mL^−1^) and glucose solution (2 mL, 100 mg·mL^−1^) were firstly mixed with GO ethanol suspension, and then silver-ammonia solution (3 mL) was added to obtain the reaction suspension. Subsequently, the reaction suspension was transferred into a 100 mL stainless autoclave. The autoclave was heated to 80 °C and pressurized by CO_2_ to 15 MPa, where a supercritical state of CO_2_ was achieved. The reaction suspension in autoclave was then subjected to vigorously magnetic agitation (600 rpm) for 1 h. After the vessel was cooled and depressurized, the black precipitates were separated by centrifugation and washed with copious deionized water and ethanol. After dried at 60 °C for 4 h in a vacuum oven, the precipitates were collected for the further characterizations and tribological tests. A similar composite as a control sample was prepared by the same fabrication process without the introduction of ScCO_2_. Silver-decorated graphene with and without ScCO_2_ were denoted as Sc-Ag/GN and Ag/GN, respectively.

### Characterization

The X-ray diffraction (XRD) patterns of the prepared nanomaterials were characterized using a Philips X’pert X-ray diffractometer (XRD, Cu-Karadiation) operating at 40 kV and 40 mA from 5° to 90°. Thermogravimetric analysis (TGA) was performed by a Netzsch Sta449C from room temperature to 600 °C at 10 °C·min^−1^ heating rate in protective nitrogen atmosphere. X-ray photoelectron spectroscopic (XPS) analysis was conducted by a Kratos Axis Ultra DLD. The morphologies of the test samples were observed with a JEOL JEM-2010F field-emission transmission electron microscope (TEM) and a JEOL JSM 6700F scanning electron microscope (SEM). The size distributions of silver particles in the Sc-Ag/GN were measured with the aid of the Image J software from the TEM images. Raman spectra of the samples were recorded by a Dilor Labram-1B multichannel confocal microspectrometer with an excitation laser of 514 nm and 20 mW. FT-IR spectra were conducted by Fourier Transform infrared spectroscopy with a Nicolet Nexus 670 FT-IR Spectrometer.

### Tribological measurement

To prepare well-dispersed and stable nano-oil samples, the synthesized nanomaterials, including GO, nano-Ag, Ag/GN and Sc-Ag/GN, were modified by stearic amine to enhance the lipophilicity. Briefly, the nanomaterials (100 mg) were dispersed in the ethanol solution of stearic amine (20 mg·mL^−1^, 20 mL) and the mixture was treated with ultrasonic processing (100 W) at 85 °C for 5 h. Then, the precipitates were washed by copious ethanol to remove the remanent modifiers. The modified additives with desirable concentration were dispersed in 10w40 engine oil by ultrasonication (80 W, 30 mins) to obtain the nano-oil samples. The tribological performances of the bare engine oil and the as-prepared nano-oils were investigated using a four-ball machine (MRS-10A, Jinan Yihua Tribology Testing Technology Co. Ltd., China). All tests were performed at loading of 343 N, rotating speed of 1200 rpm and temperature of 75 ± 1 °C for 1 h. The contact balls are GCr15 bearing steel with diameter of 12.7 mm and hardness of 61~65 HRC. Prior to the installment, the steel balls were cleaned in petroleum ether by ultrasonic cleaning and dried in air. The friction coefficients were recorded automatically by a strain sensor and the wear scar diameters (WSDs) were obtained by measuring the wear scars on the cleaned bottom balls. A 107JA optical microscopy (Shanghai Changfang Optical Instrument Co., Ltd., China) was employed to measure the WSDs and three measurements were conducted for each scar to assure the standard deviations less than 5%.

## Additional Information

**How to cite this article**: Meng, Y. *et al*. Supercritical Fluid Synthesis and Tribological Applications of Silver Nanoparticle-decorated Graphene in Engine Oil Nanofluid. *Sci. Rep.* **6**, 31246; doi: 10.1038/srep31246 (2016).

## Figures and Tables

**Table 1:** Relative atomic concentration of typical elements on the wear surfaces lubricated by 10w40+0.1 wt.% Sc-Ag/GN nano-oil after sliding for different times.

Wear scar	Atomic concentration (at. %)				
	C	O	Fe	Cr	Ag
wear scar of 5 mins	46.029	41.235	11.914	0.822	/
wear scar of 40 mins	47.402	39.838	11.736	1.024	/
wear scar of 60 mins	59.537	32.178	7.522	0.700	0.063

(343 N, 1200 rpm, 75 ± 1 °C).
